# Endobiliary Radiofrequency Ablation Combined with Gemcitabine and Cisplatin in Patients with Unresectable Extrahepatic Cholangiocarcinoma

**DOI:** 10.3390/curroncol29040182

**Published:** 2022-03-23

**Authors:** Tadahisa Inoue, Itaru Naitoh, Rena Kitano, Mayu Ibusuki, Yuji Kobayashi, Yoshio Sumida, Yukiomi Nakade, Kiyoaki Ito, Masashi Yoneda

**Affiliations:** 1 Department of Gastroenterology, Aichi Medical University, 1-1 Yazakokarimata, Nagakute 480-1195, Japan; kitano.rena.035@mail.aichi-med-u.ac.jp (R.K.); ibusuki.mayu.336@mail.aichi-med-u.ac.jp (M.I.); kobayashi.yuuji.572@mail.aichi-med-u.ac.jp (Y.K.); sumida.yoshio.500@mail.aichi-med-u.ac.jp (Y.S.); nakade.yukiomi.536@mail.aichi-med-u.ac.jp (Y.N.); kito@aichi-med-u.ac.jp (K.I.); yoneda@aichi-med-u.ac.jp (M.Y.); 2 Department of Gastroenterology and Metabolism, Nagoya City University Graduate School of Medical Sciences, 1 Kawasumi, Mizuho-cho, Mizuho-ku, Nagoya 467-8601, Japan; inaito@med.nagoya-cu.ac.jp

**Keywords:** extrahepatic cholangiocarcinoma, chemotherapy, gemcitabine, cisplatin, radiofrequency ablation, biliary stent

## Abstract

Background: Endobiliary radiofrequency ablation (RFA) is a promising treatment modality for patients with extrahepatic cholangiocarcinoma (eCCA). However, no study has investigated the combined use of endobiliary RFA and gemcitabine plus cisplatin (GC) chemotherapy. This study aimed to examine the feasibility and efficacy of endobiliary RFA with GC therapy for patients with unresectable eCCA. Methods: The study outcomes included overall survival (OS), progression-free survival (PFS), time to recurrent biliary obstruction (RBO), and adverse events associated with the treatment. These parameters were retrospectively compared between 25 patients who underwent RFA with self-expandable metal stent (SEMS) placement followed by GC therapy (with-RFA group) and a control cohort of 25 patients who underwent SEMS placement alone and GC therapy (without-RFA group). Results: The median time to RBO was significantly longer in the with-RFA group (10.7 versus 5.2 months, *p* = 0.048). The median OS was significantly higher in patients with locally advanced tumors in the with-RFA group (23.1 versus 16.6 months, *p* = 0.032), but did not differ significantly in patients with metastasis (11.4 versus 8.5 months, *p* = 0.180). Similarly, the median PFS was significantly higher in the with-RFA group in patients with locally advanced disease (10.1 versus 7.3 months, *p* = 0.015), while there was no significant difference in patients with metastasis (5.4 versus 4.4 months, *p* = 0.529). The rates of various toxicities did not differ significantly between the groups. Conclusions: Endobiliary RFA prolonged the patency period of uncovered SEMS combined with GC therapy in patients with eCCA. Although RFA also yielded survival benefits, its effect was restricted to locally advanced tumors.

## 1. Introduction

The prognosis of cholangiocarcinoma (CCA), which can be classified into intrahepatic CCA and extrahepatic CCA (eCCA) based on its location, is far poorer compared to that of most other malignancies [1,2,3,4,5]. Although surgical resection is the only curative treatment for CCA, numerous patients present with advanced disease at diagnosis, which is not eligible for surgery. Therefore, systemic chemotherapy continues to play an important role in the treatment of CCA, and gemcitabine plus cisplatin (GC) therapy has become the standard first-line regimen based on the results of a phase III randomized control trial (RCT) [6]. However, its treatment effect remains unsatisfactory since the median overall survival (OS) is reportedly only 11.7 months, necessitating further improvements and developments for the treatment of unresectable lesions.

Most patients with eCCA develop biliary strictures, which give rise to obstructive jaundice and/or cholangitis. Treating the stricture is an essential step toward the introduction and maintenance of systemic chemotherapy. Endoscopic biliary stent placement is recommended as the first-line approach since it is efficacious and less invasive, and the self-expandable metal stent (SEMS) is recommended because it possesses longer stent patency than that of plastic stents (PS) [7,8] However, recurrent biliary obstruction (RBO) occurs often despite the use of SEMS [9,10].

Endobiliary radiofrequency ablation (RFA) is a promising modality that can prolong stent patency and survival in patients with eCCA [11,12] and whose application has reportedly increased worldwide in recent years. However, to the best of our knowledge, no study has investigated the combined use of endobiliary RFA and GC therapy. The current study aimed to examine the tolerability, feasibility, and efficacy of endobiliary RFA with GC therapy in patients with unresectable eCCA.

## 2. Patients and Methods

### 2.1. Study Design and Patients

This retrospective, comparative, multi-center study enrolled 25 consecutive patients, aged 20 years or above, with unresectable eCCA and biliary obstruction, who underwent endobiliary RFA and GC therapy as first-line chemotherapy between 2016 and 2021. The exclusion criteria were as follows: malignancies that were not proven pathologically, short strictures that were unsuitable for the RFA catheter used in the study [12], technical failure of transpapillary drainage, and patients who refused to participate in the study. All patients underwent pre-drainage with a PS or nasobiliary drainage at the time of forceps biopsy with/without brush cytology for the stricture. After confirmation of the pathological malignancy and unresectability, including metastasis, extensive intraepithelial spread/progression, and patient’s general condition, endobiliary RFA with SEMS placement were performed, followed by the implementation of GC therapy. The outcomes were compared with those of a historical control group consisting of an equal number of consecutive patients who underwent GC therapy and SEMS placement without RFA, before the RFA was introduced at the institutions.

The institutional review board of Aichi Medical University Hospital approved this study, which was conducted in accordance with the principles of the Declaration of Helsinki (approval number: 2020-149). All patients were offered the option to opt out of the study if they did not want to have their data published.

### 2.2. Endobiliary RFA with SEMS Placement

**Figure 1:**
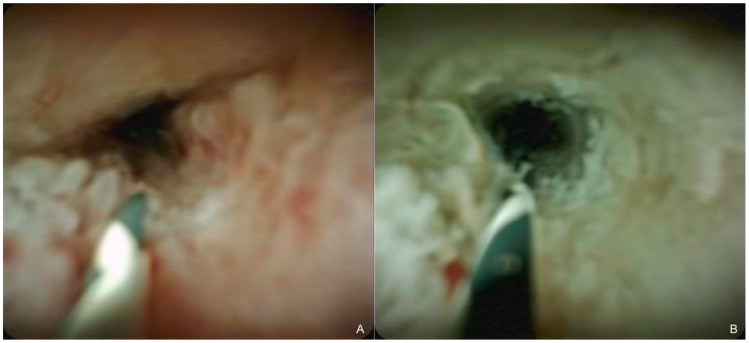
Cholangioscopic findings of the biliary stricture before (**A**) and after (**B**) radiofrequency ablation. The tumor showed coagulative necrosis, and the stricture improved after the ablation procedure.

A standard side-viewing duodenoscope (Olympus Medical Systems, Tokyo, Japan) was inserted into the duodenum, and biliary cannulation was performed using a standard catheter and guidewire. The guidewire was advanced thorough the stricture, followed by the insertion of an RFA catheter (Habib Endo HPB; Boston Scientific, Marlborough, MA, USA) over the guidewire, and subsequently, the stricture was ablated for 90 s with a power of 7–10 W, which was delivered by a VIO300D generator (ERBE Elektromedizin GmbH, Tübingen, Germany) (Figure 1). If the length of the stricture was greater than the ablation scope of a single RFA application or if the stricture extended to the right and left hepatic ducts, RFA application was repeated until the entire length of the stricture was ablated, while ensuring minimal overlapping [13]. After ablation, an uncovered SEMS was deployed across the stricture; bilateral deployment using stent-by-stent technique was basically performed in patients with hilar strictures. SEMS diameters were basically 10 mm for distal strictures and 8 mm for hilar strictures, and the length of SEMS was chosen according to the length of the strictures. Some patients received repeated RFA in the event of RBO. An uncovered SEMS was deployed in the same manner without RFA in the control group.

### 2.3. GC Therapy

Each chemotherapy cycle comprised gemcitabine (1000 mg/m^2^) and cisplatin (25 mg/m^2^), which was administered on days 1 and 8, followed by 1 week of rest. The dose was modified based on the incidence of adverse events associated with the treatments and/or the physician/patient discretion. GC therapy was continued until disease progression, occurrence of unacceptable toxic effects, or if the patient refused to continue with treatment. RBO was not considered to constitute disease progression. Second-line chemotherapy or best supportive care was initiated in the event of disease progression.

### 2.4. Follow-Up

All patients were followed up using examinations and laboratory tests, including the monitoring of tumor markers and computed tomography, which was performed at least once in 2–3 months to evaluate disease progression. Computed tomography was also performed when RBO was suspected.

### 2.5. Outcomes and Definitions

The study outcomes included OS, progression-free survival (PFS), best response, adverse events associated with treatment, and the clinical success and incidence of RBO associated with SEMS placement with and without RFA. The outcomes were compared between the treatment cohort that underwent SEMS placement and RFA with GC therapy (with-RFA group) and the control cohort that underwent SEMS placement alone with GC therapy (without-RFA group).

The OS was measured from the date of initiation of GC therapy to the date of the patient’s death, and PFS was measured from the date of initiation of GC therapy until either disease progression or the patient’s death. The best response was judged according to the Response Evaluation Criteria in Solid Tumors version 1.1 [14]. Among the patients who showed no metastasis, lesions in which evaluation became difficult owing to the presence of SEMS were classified as non-measurable lesions. Adverse events were assessed and graded according to the Common Terminology Criteria of Adverse Events version 5.0. RBO was defined as the recurrence of jaundice and/or cholangitis along with biliary dilation on imaging studies, and the time to RBO was defined as the period from the date of performing SEMS placement until the date of occurrence of RBO [15].

### 2.6. Statistical Analysis

Fisher’s exact test was used to evaluate the differences between the categorical variables, and the Mann–Whitney U test was used to compare the continuous variables. The Kaplan–Meier method was used to estimate the OS, PFS, and time to RBO, and the curves were compared using the log-rank test. The data of patients in whom RBO did not occur until the time of death were considered to be censored. All statistical analyses were conducted using EZR version 1.54 (Saitama Medical Centre, Jichi Medical University, Saitama, Japan) [16]. *p* values < 0.05 were considered to indicate statistical significance.

## 3. Results

### 3.1. Patient Characteristics

Table 1 depicts the baseline characteristics of the study population. All patients had eCCA, and all the lesions were identified as adenocarcinomas on histopathological examination. There were no characteristic values that differed significantly between the groups. The tumors were located in the hilar region in 84% (21/25) of patients in the with-RFA group and 88% (22/25) of patients in the without-RFA group. Metastases were observed in 48% (12/25) of patients in the with-RFA group and 60% (15/25) of patients in the without-RFA group. There were no differences between the two groups with regard to factors related to SEMS, including deployment method.

### 3.2. SEMS Placement with/without Endobiliary RFA

**Figure 2:**
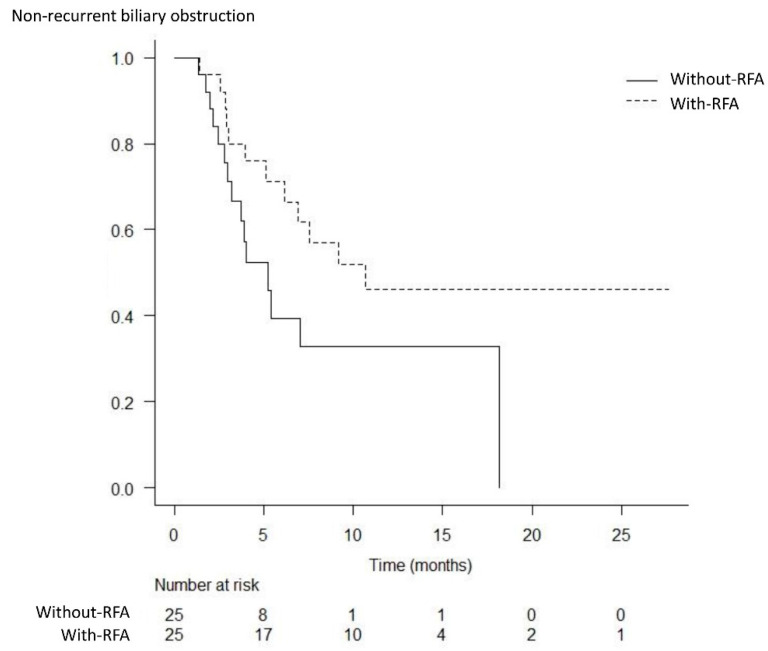
Kaplan–Meier analysis of the time to recurrent biliary obstruction. The median time to recurrent biliary obstruction was significantly longer in the with-RFA group than that in the without-RFA group (10.7 months versus 5.2 months, *p* = 0.048).

Table 2 depicts the outcomes of SEMS placement with and without RFA. The clinical success rate of the procedure was 100% (25/25) in both groups. The rate of procedure-related adverse events other than RBO was 8% (2/25) in both groups. RBO occurred in 44% (11/25) of patients in the with-RFA group and 60% (15/25) of patients in the without-RFA group (*p* = 0.571), while the median time to RBO was significantly longer in the with-RFA group (10.7 months versus 5.2 months, *p* = 0.048) (Figure 2).

### 3.3. Treatment Exposure

The mean frequency of GC therapy was 8.2 cycles (range: 1–22 cycles) and 7.1 cycles (range: 1–20) in the with-RFA and without-RFA groups, respectively, which lacked a statistically significant difference (*p* = 0.408). The relative dose intensity was 83% and 81% for gemcitabine and cisplatin in the with-RFA group, and 87% and 80% in the without-RFA group, respectively, without significant differences (*p* = 0.533 and *p* = 0.439). Second-line chemotherapy was performed in 40% (10/25) of patients in the with-RFA group and 40% (10/25) of patients in the without-RFA group (*p* = 1.000). The mean frequency of RFA sessions was 1.84 times (range: 1–4 times) in the with-RFA group.

### 3.4. Efficacy

**Figure 3:**
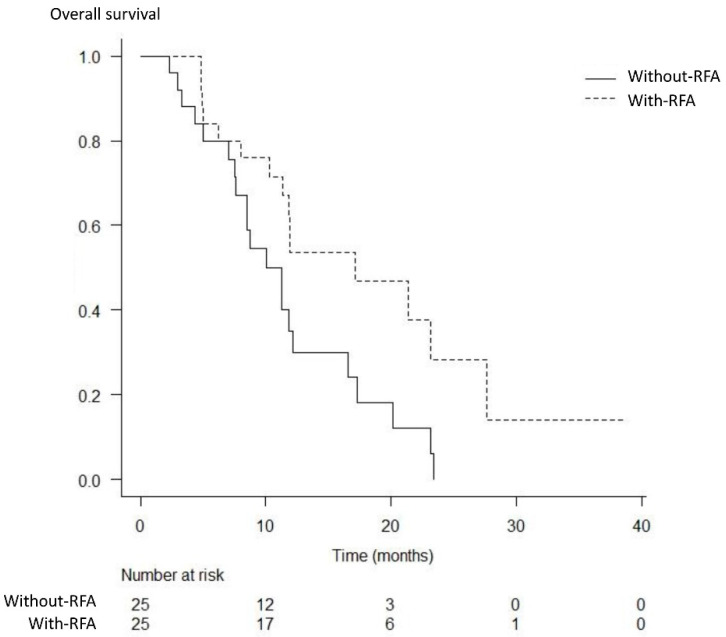
Kaplan–Meier analysis of the overall survival. The median overall survival was significantly higher in the with-RFA group than that in the without-RFA group (17.1 months versus 11.3 months, *p* = 0.017).

**Figure 4:**
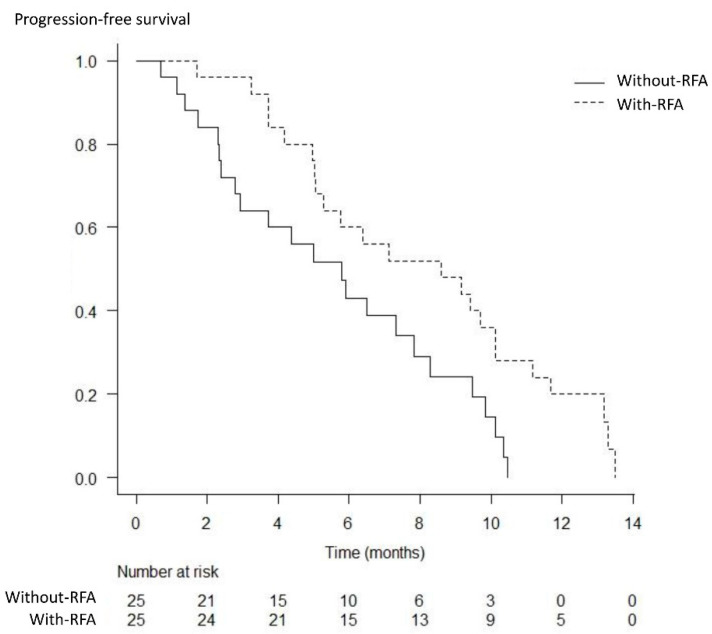
Kaplan–Meier analysis of the progression-free survival. The median progression-free survival was significantly higher in the with-RFA group than that in the without-RFA group (8.6 months versus 5.8 months, *p* = 0.014).

**Figure 5:**
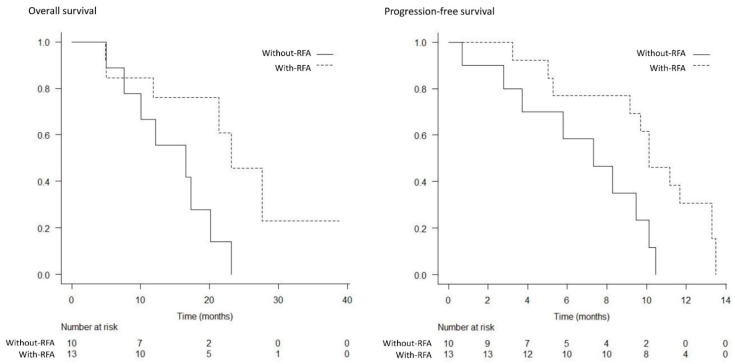
Kaplan–Meier analysis of the overall survival and progression-free survival in patients with locally advanced tumors. The median overall survival (23.1 months versus 16.6 months, *p* = 0.032), and progression-free survival (10.1 months vs. 7.3 months, *p* = 0.015) were significantly higher in the with-RFA group compared to those of the without-RFA group.

**Figure 6:**
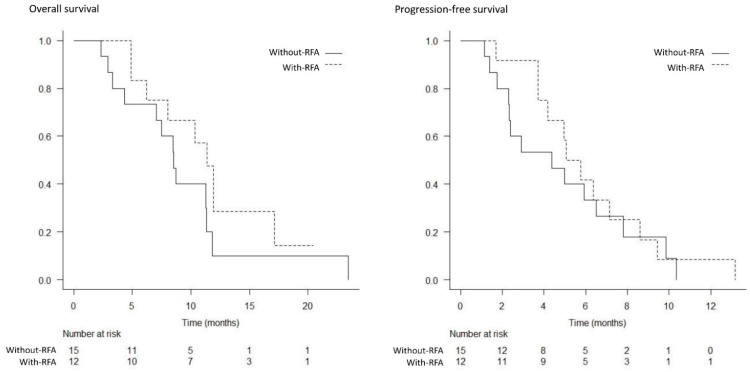
Kaplan–Meier analysis of the overall survival and progression-free survival in patients with metastasis. There were no significant differences in the median overall survival (11.4 months versus 8.5 months, *p* = 0.180) and progression-free survival (5.4 months versus 4.4 months, *p* = 0.529), between the with-RFA and without-RFA groups.

The best response was measurable in 22 patients in the with-RFA group and 23 patients in the without-RFA group. A complete response was not achieved in either group. The partial response, stable disease, and progressive disease rates were 18% (4/22), 64% (14/22), and 18% (4/22) in the with-RFA group, and 17% (4/23), 57% (13/23), and 26% (6/23) in the without-RFA group, respectively; thus, significant differences were not observed between the two groups (*p* = 1.000, *p* = 0.763, and *p* = 0.722) (Table 3). The median OS and PFS were significantly higher in the with-RFA group from among the total study population (17.1 months versus 11.3 months, *p* = 0.017; 8.6 months versus 5.8 months, *p* = 0.014) (Figure 3 and Figure 4). The median OS and PFS were significantly higher in the with-RFA group in patients with locally advanced tumors (23.1 months versus 16.6 months, *p* = 0.032; 10.1 months versus 7.3 months, *p* = 0.015) (Figure 5), while they did not differ significantly between the two groups in patients with metastasis (11.4 months versus. 8.5 months, *p* = 0.180; 5.4 months versus 4.4 months, *p* = 0.529) (Figure 6).

### 3.5. Safety

The details of grade 3 or higher adverse events are presented in Table 4. Neutropenia was the most frequently observed adverse event in both groups, occurring in 52% (13/25) and 44% (11/25) patients in the with-RFA and without-RFA groups, respectively. The rate of any hematological toxicity did not differ significantly between the groups. There were also no significant differences in the rate of any non-hematological toxicities (besides RBO) between the groups. Treatment-related deaths did not occur in either group.

## 4. Discussion

The present study demonstrated that endobiliary RFA prolonged the time to RBO when used concomitantly with uncovered SEMS, followed by GC therapy. Moreover, RFA showed an additive survival prolonging effect on GC therapy for eCCA but was limited to patients with locally advanced tumors.

The treatment and management of biliary strictures is a critical aspect of the treatment of eCCA, especially in patients who have undergone chemotherapy. RBO is often accompanied by sequelar cholangitis, which can be serious in patients who are in a state of myelosuppression following chemotherapy. Moreover, reintervention and hospitalization are required in the event of RBO, which can cause deterioration in the patient’s quality of life, while chemotherapy, which is directly related to survival, also needs to be stopped due to RBO. The SEMS possesses longer stent patency compared to PS, and recent guidelines recommend its deployment for unresectable cases [7]. Moreover, some studies, including a meta-analysis, reported that SEMS contributed not only to stent patency, but also to survival [17,18]. However, the stent patency period is insufficient, and RBO often occurs despite SEMS deployment, rendering the extension of stent patency an extremely important theme of research in the current milieu.

Endobiliary RFA was first developed with the goal of extending the duration of stent patency [19]. However, previous studies have demonstrated conflicting results, making its utility controversial [12]. The primary reason for this ambiguity can be attributed to the fact that crucial factors, such as the primary cancer type, disease stage, and type of stent, were not uniform in most previous studies. The present study showed that endobiliary RFA prolonged the patency period of uncovered SEMS in the setting where GC therapy was performed for eCCA.

Studies have also suggested that endobiliary RFA may prolong survival in patients with eCCA via tumor volume reduction associated with coagulative necrosis, superior stent patency, and antitumor immunity [20,21,22,23]. Recently, two RCTs compared stenting combined with RFA with stenting alone in patients with eCCA. The first study by Yang et al. [24] reported that the mean survival time was significantly longer in the RFA with stent group (13.2 versus 8.3 months, *p* < 0.001), and the other study by Gao et al. [25] also reported that the median cumulative survival was significantly longer in the RFA with stent group (14.3 versus 9.2 months *p* < 0.001). However, patients who underwent chemotherapy were excluded from the Yang et al. study population, while their number was considerably lower in the Gao et al. study population. Yang et al. [26] later conducted another study that compared RFA with stenting and S-1 chemotherapy versus RFA with stenting for eCCA and reported that the median OS was longer in the group that underwent RFA combined with S-1 (16.0 versus 11.0 months, *p* < 0.001). Therefore, the results of these studies showed that RFA is expected to exert a considerable effect on the survival in patients with eCCA, and chemotherapy is also anticipated to confer an additional survival benefit on RFA. However, to date, no study has verified the additive effect of RFA on chemotherapy for eCCA or investigated the combination of RFA with GC therapy, which is the standard first-line regimen for eCCA. The present study ascertained that the combination of RFA with GC therapy yielded longer survival compared to those of GC therapy alone.

However, the present study demonstrated that the significantly longer OS associated with RFA was only observed in the patient cohort with locally advanced tumors. This observation may indicate the survival benefit conferred by endobiliary RFA, whose influence is limited to eCCA without metastasis. Future studies are required to evaluate the systemic effect of endobiliary RFA in greater detail. Moreover, the appropriate number of RFA sessions required to achieve an adequate effect is unclear. Although several studies conducted multiple sessions of RFA, the additional effects of multiple RFA sessions, criteria determining the indications for the need of additional ablation, and the requisite frequency for ablation remain uncertain. The present study performed RFA at the time of initial SEMS placement and RBO occurrence, and the mean frequency of the RFA sessions was 1.84, but the appropriateness of this regimen is also unclear. These aspects unveil important issues and considerations for future studies.

Patient selection is another important consideration for RFA. The RFA catheter used in the present study was a bipolar device with two 8 mm ring electrodes placed 8 mm apart at the tip of the catheter. A sufficient ablative effect could not be obtained in some cases, such as those with short-length strictures, because the two electrodes had to be maintained in proper contact with the stricture tissue [13,27]. Although a recently developed catheter with a shorter distance between the electrodes may mitigate this limitation [28,29], further research and improvement of devices are essential [30] for endobiliary RFA to be standardized as a treatment modality and for its widespread use.

The present study had several limitations. First, the study was retrospective and nonrandomized in design, and thus, the possibility of the resulting selection bias cannot be excluded. The results might have overestimated the utility of RFA because it may have been avoided in difficult cases, such as complicated hilar strictures. Second, the sample size was small. Although there were no significant differences in the several values and outcomes, it is possible that the study was statistically underpowered. Third, all endobiliary RFA procedures were conducted by endoscopists with experience in performing endoscopic biliary stenting and endobiliary RFA. Since RFA is only visible under the fluoroscopy during the procedure, some of the evidence on the ablative effect achieved and safety associated with RFA may be empirical in nature. It was not possible to ascertain whether all the lesions were well ablated or not because not all patients underwent cholangioscopy after ablation in the present study.

## 5. Conclusions

Despite these limitations, this was the first study that focused on the combination of RFA and SEMS placement with GC therapy for patients with unresectable eCCA, which yielded promising results, especially in cases with locally advanced tumors. This study provided important evidence for the role of endobiliary RFA in treating eCCA and further enhancing the outcomes. Further clinical trials should be performed on the basis of these results.

## Figures and Tables

**Table 1:** Patients’ baseline characteristics.

		With-RFA Group	Without-RFA Group	*p* Value
Number of patients, *n*		25	25	
Sex (male/female), *n*		10/15	17/8	0.088
Mean age, years (range)		78 (50–86)	74 (48–85)	0.073
ECOG performance status, *n* (%)				0.242
	0	9 (36)	12 (48)	
	1	13 (52)	13 (52)	
	2	3 (12)	0	
Diagnosis, *n* (%)				
	Extrahepatic cholangiocaricinoma (adenocarcinoma)	25 (100)	25 (100)	1.000
Location of tumor, *n* (%)				1.000
	Hilar	21 (84)	22 (88)	
	Distal	4 (16)	3 (12)	
Metastatic, *n* (%)		12 (48)	15 (60)	0.571
Metastatic site ^†^, *n* (%)				
	Peritoneum	6 (24)	2 (8)	0.247
	Liver	5 (20)	5 (20)	1.000
	Lymph nodes	4 (16)	5 (20)	1.000
	Bone	1 (4)	4 (16)	0.349
	Lung	0	4 (16)	0.110
	Adrenal	0	1 (4)	1.000
Cholangitis ^‡^, *n* (%)		5 (20)	3 (12)	0.702
Mean bilirubin level ^‡^, mg/dL (range)		1.76 (0.41–20.74)	2.69 (0.31–18.60)	0.146
Mean alkaline phosphatase level ^‡^, U/L (range)		864 (276–3772)	888 (197–3372)	0.091
Mean CEA, ng/mL		10.1 (1–862)	9.8 (1–1120)	0.745
Mean CA19-9, U/mL (range)		1086 (2–140054)	912 (2–152510)	0.564

ECOG, Eastern Cooperative Oncology Group. ^†^ There are some overlaps between the sites. ^‡^ Before pre-drainage.

**Table 2:** Outcomes of stent placement with/without radiofrequency ablation.

		With-RFA Group	Without-RFA Group	*p* Value
Clinical success, *n* (%)		25/25 (100)	25/25 (100)	1.000
Procedure-related adverse events besides RBO, *n* (%)		2/25 (8)	2/25 (8)	1.000
	Cholangitis	1	1	
	Pancreatitis	1	0	
	Bleeding	0	1	
Incidence of RBO, *n* (%)		12/25 (44)	15/25 (60)	0.571
Median time to RBO, months (95% CI)		10.7 (5.1-NA)	5.2 (3.0-NA)	0.048
	6-month non-RBO rate	71%	39%	
	1-year non-RBO rate	46%	33%	

RBO, recurrent biliary obstruction; CI, confidence interval.

**Table 3:** Efficacy of gemcitabine plus cisplatin chemotherapy with/without radiofrequency ablation.

		With-RFA Group	Without-RFA Group	*p* Value
Best overall response, *n* (%)				
	Complete response	0	0	1.000
	Partial response	4/22 (18)	4/23 (17)	1.000
	Stable disease	14/22 (64)	13/23 (57)	0.763
	Progressive disease	4/22 (18)	6/23 (26)	0.722
Median overall survival, months (95% CI)				
	Total population	17.1 (10.3–27.6)	11.3 (7.5–12.1)	0.017
	Locally advanced	23.1 (11.8–NA)	16.6 (5.0–20.1)	0.032
	Metastatic	11.4 (4.9–17.1)	8.5 (3.3–11.3)	0.180
Progression-free survival, months (95% CI)				
	Total population	8.6 (5.1–10.1)	5.8 (2.8–7.8)	0.014
	Locally advanced	10.1 (5.3–13.3)	7.3 (0.7–10.1)	0.015
	Metastatic	5.4 (3.7–8.6)	4.4 (1.7–6.5)	0.529

CI, confidence interval.

**Table 4:** Grade 3 or higher adverse events.

	With-RFA Group	Without-RFA Group	*p* Value
Hematological toxicities, *n* (%)			
Anemia	8/25 (32)	7/25 (28)	1.000
Thrombocytopenia	5/25 (20)	7/25 (28)	0.742
Leukopenia	8/25 (32)	8/25 (32)	1.000
Neutropenia	13/25 (52)	11/25 (44)	0.778
Febrile neutropenia	0	0	
Non-hematological toxicities *, *n* (%)			
Biliary tract infection	2/25 (8)	1/25 (4)	1.000
Appetite loss	1/25 (4)	0	1.000
Urticaria	1/25 (4)	1/25 (4)	1.000
Constipation	0	1/25 (4)	1.000

* Excluded events related to recurrent biliary obstruction.

## Data Availability

Not applicable.
